# Scalable Graphene–MoS_2_ Lateral Contacts for High-Performance 2D Electronics

**DOI:** 10.3390/ma18204689

**Published:** 2025-10-13

**Authors:** Woonggi Hong

**Affiliations:** Department of Convergence Semiconductor Engineering, Dankook University, 152 Jukjeon-ro, Suji-gu, Yongin-si 16890, Gyeonggi-do, Republic of Korea; woonggi.hong@dankook.ac.kr

**Keywords:** graphene, molybdenum disulfide (MoS_2_), heterostructure, field-effect transistors, contact properties

## Abstract

As the scaling of silicon-based CMOS technology approaches its physical limits, two-dimensional (2D) materials have emerged as promising alternatives for future electronic devices. Among them, MoS_2_ is a leading candidate due to its fascinating semiconducting nature and compatibility with CMOS processes. However, high contact resistance at the metal–MoS_2_ interface remains a major bottleneck, limiting device performance. In this study, we report the fabrication and characterization of graphene–MoS_2_ (Gr–MoS_2_) lateral heterostructure FETs, where monolayer graphene, synthesized by inductively coupled plasma chemical vapor deposition (ICP-CVD), is directly used as the source and drain. Bilayer MoS_2_ is selectively grown along graphene edges via edge-guided CVD, forming a chemically bonded in-plane junction without transfer steps. Electrical measurements reveal that the Gr–MoS_2_ FETs exhibit a threefold increase in average field-effect mobility (3.9 vs. 1.1 cm^2^ V^−1^ s^−1^) compared to conventional MoS_2_ FETs. Y-function analysis shows that the contact resistance is significantly reduced from 85.8 kΩ to 20.5 kΩ at V_G_ = 40 V. These improvements are attributed to the replacement of the conventional metal–MoS_2_ contact with a graphene–metal contact. Our results demonstrate that lateral heterostructure engineering with graphene provides an effective and scalable strategy for high-performance 2D electronics.

## 1. Introduction

The continuous scale-down of semiconductor devices has approached fundamental physical and materials limits within traditional silicon-based complementary metal-oxide semiconductor (CMOS) platforms [1]. In response, two-dimensional (2D) materials have garnered significant interest as promising channel and contact materials owing to their atomically thin geometry and rich diversity in electronic properties [2,3,4]. Among them, semiconducting transition metal dichalcogenides (TMDs), particularly molybdenum disulfide (MoS_2_), have emerged as leading candidates due to their sizable bandgap, high on/off current ratio, and compatibility with CMOS processing [5,6,7,8]. However, one of the key bottlenecks in TMDs-based field-effect transistors (FETs) lies in the high contact resistance at the metal–semiconductor junction [9,10,11,12]. The absence of strong chemical bonding at the 2D material surface, combined with Fermi level pinning, often leads to the formation of significant Schottky barriers that inhibit efficient carrier injection [13,14]. Various approaches have been explored to mitigate this issue, including phase engineering [15,16], work function modulation [17,18], and the introduction of interfacial layers [19,20]. Despite these advances, scalable and reproducible contact engineering strategies remain limited. In this context, graphene has been proposed as a practical alternative to conventional metals for contacting 2D semiconductors [4,21,22,23]. Its work function tunability, high electrical conductivity, and atomically smooth surface make it an ideal interfacial material that can potentially form low-barrier van der Waals (vdW) contacts with semiconducting TMDs. In particular, laterally connected graphene–MoS_2_ (Gr–MoS_2_) heterostructures, fabricated via bottom-up chemical vapor deposition (CVD), offer a clean, scalable platform for seamless integration without the need for transfer or lithographic alignment steps [24,25,26]. Several studies have demonstrated that lateral Gr–MoS_2_ heterostructures offer superior electrical characteristics over conventional MoS_2_ FETs, largely attributed to the formation of low-resistance, atomically sharp Gr–MoS_2_ interfaces [27,28,29].

Building upon these results, the present study systematically investigates the role of graphene contacts in lateral heterostructure-based FETs by quantitatively analyzing their resistance components and comparing them against those of conventional MoS_2_ FETs. By utilizing identical growth and device fabrication conditions, we isolate the impact of contact geometry on device performance. We further explore the effect of channel length scaling and provide direct comparisons of contact and channel resistance components. To the best of our knowledge, a systematic and quantitative decomposition of resistance contributions in Gr–MoS_2_ heterostructure FETs under strictly identical fabrication conditions has not been reported. This direct side-by-side comparison with conventional MoS_2_ FETs enables us to clearly isolate the intrinsic effect of the Gr–MoS_2_ lateral interface on device performance. By employing the Y-function method to separately evaluate contact and channel resistances, our study provides new insight into the role of graphene contacts that goes beyond the general understanding that graphene reduces Schottky barriers. These results highlight the effectiveness of the Gr–MoS_2_ lateral interface in optimizing parasitic resistances and enhancing carrier transport, emphasizing its potential for future nanoscale electronics and contact-limited device platforms.

## 2. Experimental Section

### 2.1. Device Fabrication Process

Following the growth process, both the MoS_2_–graphene lateral heterostructure and reference MoS_2_ samples were coated with a negative photoresist to define the device structure. Source and drain regions were patterned using a photolithography process. For metallization, Pd (15 nm)/Au (35 nm) was deposited on the heterostructure devices, while Ti (15 nm)/Au (35 nm) was used for the MoS_2_-only devices, both via thermal evaporation under a high vacuum of approximately 10^−6^ Torr. Subsequent lithography was performed to define the channel region, and unneeded MoS_2_ outside the active area was removed using oxygen plasma etching (ICP-asher, Korea Vacuum Tech Co., LTD, Gimpo-si, Gyeonggi-do, Republic of Korea). The fabricated devices featured channel lengths and widths ranging from a few micrometers to several tens of micrometers.

### 2.2. Electrical Property Characterization

Before electrical measurements, all fabricated devices were annealed at 250–300 °C for 2 h under high vacuum (~10^−6^ Torr) to eliminate residual moisture on the channel surface. Electrical characterization was carried out using a semiconductor parameter analyzer (4200 SCS, Keithley Instruments, Cleveland, OH, USA) in conjunction with a probe station (MS-TECH). All measurements were performed under vacuum conditions (~50 mTorr) and in the dark to minimize the effects of ambient gas adsorption and photogenerated electrons.

## 3. Results and Discussion

**Figure 1:**
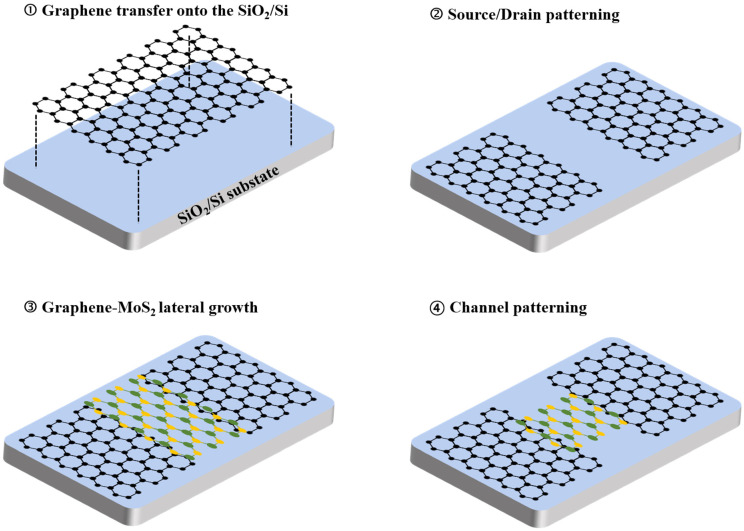
Schematic illustration of the fabrication process for a graphene–MoS_2_ lateral heterostructure.

The overall fabrication process of the graphene–MoS_2_ (Gr–MoS_2_) lateral heterostructure is schematically illustrated in Figure 1. Monolayer graphene was first synthesized on a four-inch SiO_2_/Si wafer coated with a 300 nm-thick Cu film using inductively coupled plasma chemical vapor deposition (ICP-CVD). A gas mixture of C_2_H_2_ (1 sccm) and Ar (100 sccm) was introduced into the chamber under 50 W RF plasma at 980 °C to promote monolayer graphene growth [30,31]. After synthesis, the graphene was transferred onto a SiO_2_ (90 nm)/Si substrate via a metal-etching-free transfer method, which avoids contamination and prevents structural damage to the graphene layer during transfer [30]. Photolithography and oxygen plasma etching were then employed to define patterned graphene regions, selectively exposing windows for subsequent MoS_2_ growth. MoS_2_ was synthesized using atmospheric pressure chemical vapor deposition (APCVD) in a four-inch tube furnace equipped with two independently controlled heating zones. MoO_3_ (Sigma Aldrich, St. Louis, MO, USA, ≥99.5%) and sulfur (Sigma Aldrich, 99.98%) powders were placed in alumina crucibles at the downstream (700 °C) and upstream (320 °C) regions, respectively. The growth proceeded under a 100 sccm Ar flow for 30 min, and perylene-3,4,9,10-tetracarboxylic acid tetrapotassium (PTAS) was used as a seeding promoter to facilitate nucleation. To favor lateral rather than vertical heterostructure formation, the MoS_2_ growth temperature was carefully optimized to 700 °C, a regime known to suppress MoS_2_ nucleation on the graphene basal plane while promoting edge-selective lateral growth [32]. The graphene edges served as nucleation sites, enabling MoS_2_ to grow laterally from both sides of the patterned graphene. As growth progressed, the MoS_2_ domains coalesced between adjacent graphene regions, forming a continuous semiconductor channel laterally stitched to the graphene source and drain electrodes. Unlike conventional vertical stacking methods, this in-plane heterostructure offers a chemically bonded interface and facilitates scalable, transfer-free fabrication, making it suitable for large-area integration. For field-effect transistor (FET) fabrication, photolithography was used to define the channel region, and the MoS_2_ outside the channel region was removed by O_2_ plasma etching, ensuring that only the MoS_2_ within the channel area remained.

**Figure 2:**
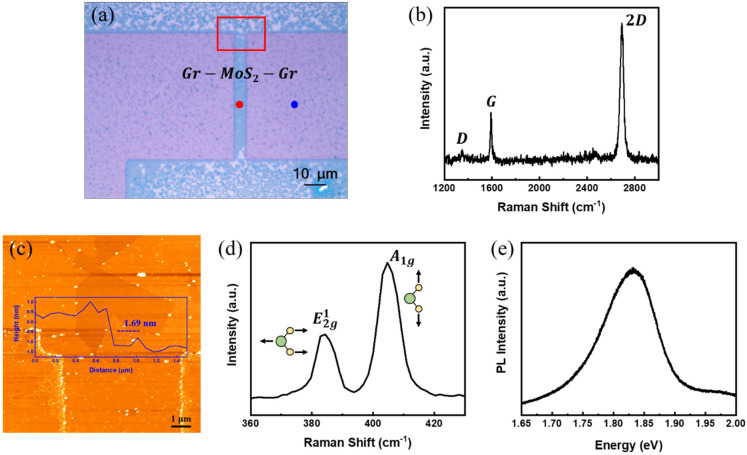
(**a**) Optical microscope (OM) image of the as-grown graphene–MoS_2_ lateral heterostructure. (**b**) Raman spectrum of graphene measured at the blue-marked point in (**a**). (**c**) Atomic force microscopy (AFM) image of the red-boxed region in (**a**), confirming the MoS_2_ thickness to be approximately 1.69 nm. (**d**) Raman spectrum and (**e**) photoluminescence (PL) of MoS_2_, both acquired at the red-marked point in (**a**).

To verify the structural integrity and layer thickness of the synthesized MoS_2_ within the lateral heterostructure, we conducted a series of morphological and spectroscopic characterizations. Figure 2a shows an optical microscope (OM) image of a representative Gr–MoS_2_ lateral heterostructure where the MoS_2_ is contacted laterally by graphene edges. As shown in Figure 2b, the Raman spectrum of the graphene film, measured at the blue-marked point in Figure 2a, exhibits a prominent 2D peak with a 2D/G intensity ratio greater than 2 and a negligible D peak, confirming the monolayer thickness and high crystalline quality of the synthesized graphene [33]. The red box indicates the region selected for atomic force microscopy (AFM) analysis. The AFM image, shown in Figure 2c, confirms the formation of a uniform MoS_2_ film at the heterointerface, with the measured step height of approximately 1.69 nm. Although the theoretical thickness of monolayer MoS_2_ is ~0.62 nm, the AFM measurements typically yield ~0.8 nm per layer due to substrate effects and surface adsorbates [34,35,36], making the observed step height consistent with a bilayer film. To evaluate the structure and crystallographic properties of the MoS_2_ domain, Raman spectroscopy was performed at the red-marked point in Figure 2a. As shown in Figure 2d, the characteristic Raman peaks of MoS_2_, corresponding to the in-plane $E_{2g}^{1}$ mode and the out-of-plane $A_{1g}$ mode, appear at ~384.4 and ~404.7 cm^−1^, respectively. The positions of these peaks are consistent with the semiconducting 2H phase of MoS_2_ [37], further confirming the structural integrity of the synthesized layer. The peak separation (~20.3 cm^−1^) further confirms that the MoS_2_ is in the bilayer regime [34,38], in good agreement with AFM results. Photoluminescence (PL) spectroscopy was also conducted to assess the optical quality of the MoS_2_ layer. The spectrum in Figure 2e exhibits a pronounced A-exciton emission peak near 1.83 eV, showing a sharp and intense PL response that indicates high optical quality with minimal defect-related nonradiative recombination. Collectively, these structural and optical characterizations verify the successful formation of a bilayer MoS_2_ channel laterally stitched to graphene, forming a well-defined, clean, and electronically functional in-plane heterojunction suitable for FET fabrication.

**Figure 3:**
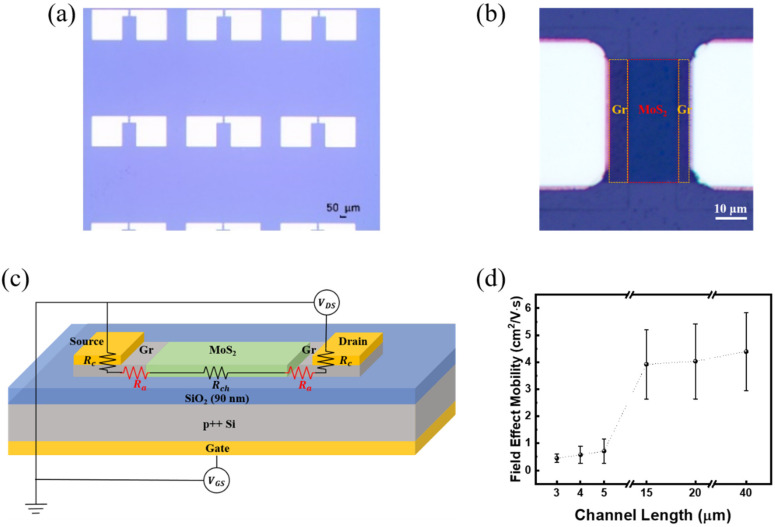
(**a**) Optical microscope (OM) image of the fabricated device array based on graphene–MoS_2_ lateral heterostructures. (**b**) Enlarged OM image of a representative device, showing the MoS_2_ channel region contacted by graphene. (**c**) Schematic illustration of the device structure and resistance components, including the contact resistance ($R_{c}$), access resistance ($R_{a}$), and channel resistance ($R_{ch}$). (**d**) Field-effect mobility as a function of MoS_2_ channel length, showing a decreasing trend with shorter channels due to the increasing influence of access resistance.

To evaluate the electrical properties of the Gr–MoS_2_ lateral heterostructure, we fabricated back-gated field-effect transistors (FETs). Figure 3a shows an OM image of the fabricated device array, where multiple devices with varying channel lengths and widths were prepared to facilitate statistical transport analysis. A representative single device is shown in Figure 3b, highlighting the laterally stitched MoS_2_ channel region (red dashed region) between two patterned graphene (Gr) source and drain (yellow dashed regions). The channel geometry was precisely defined by photolithography and O_2_ plasma etching, and Pd (15 nm)/Au (35 nm) contact metals were deposited on graphene to ensure low-resistance ohmic contact [39]. A schematic of the fabricated FET and its associated electrical resistance components is illustrated in Figure 3c. The total resistance between the source and drain electrodes consists of three major components: (i) the contact resistance ($R_{c}$) between metal and graphene; (ii) the access resistance ($R_{a}$) arising from the Gr–MoS_2_ transition region, which is less effectively modulated by the back-gate field; and (iii) the intrinsic channel resistance ($R_{ch}$) of the MoS_2_ layer. This decomposition allows for a detailed assessment of how contact engineering and channel length scaling influence overall device behavior. To investigate the scaling effects, we extracted the field-effect mobility ($\mu_{FE}$) as a function of channel length, as shown in Figure 3d. The mobility was calculated using the transconductance in the linear regime of the transfer curve. $\mu_{FE}$ exhibits a clear decreasing trend with decreasing channel length. Since the graphene regions corresponding to the access resistance ($R_{a}$) are fixed at 5 µm in length, their relative contribution becomes more significant as the MoS_2_ channel length decreases. This result highlights the importance of carefully engineering the contact and transition regions, especially for scaled devices, where parasitic resistances can severely degrade performance. To minimize the influence of access resistance and ensure accurate extraction of intrinsic transport properties, our analysis was limited to devices with MoS_2_ channel lengths greater than 15 µm.

**Figure 4:**
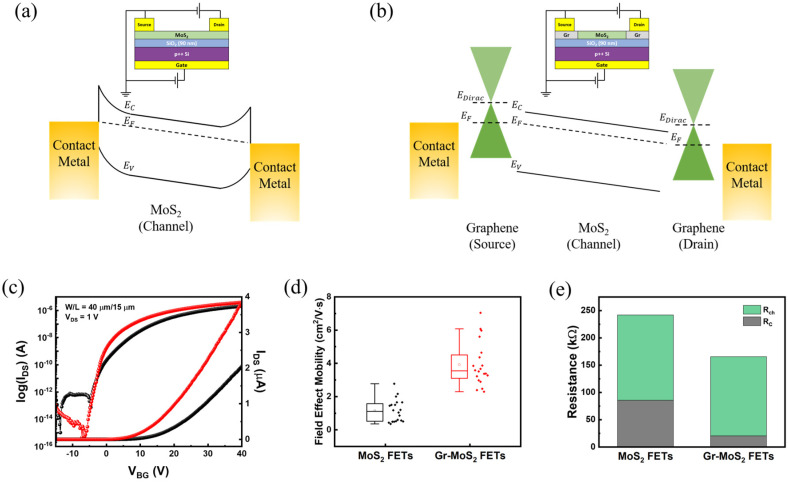
Schematic illustration of (**a**) a conventional MoS_2_ FET structure and (**b**) Gr–MoS_2_ FET, along with their corresponding energy band diagrams under positive gate and drain bias. (**c**) Transfer characteristics of MoS_2_ FETs (black) and Gr–MoS_2_ FETs (red). (**d**) Extracted field-effect mobility values for both FET types. (**e**) Comparison of the contact and channel resistance components in MoS_2_ and Gr–MoS_2_ FETs.

To assess the impact of contact engineering using graphene, we fabricated and compared conventional MoS_2_ FETs and Gr–MoS_2_ lateral heterostructure FETs. Figure 4a,b schematically illustrate the device architectures and corresponding band alignments under positive gate and drain bias. In conventional MoS_2_ FETs, metal–semiconductor interfaces typically suffer from Fermi-level pinning [11,40] and large Schottky barriers [41,42], which hinder efficient carrier injection. In contrast, the Gr–MoS_2_ FET structure replaces the conventional metal–MoS_2_ contact with a metal–graphene interface, in which graphene serves as an atomically thin, highly conductive bridge between the metal electrode and the MoS_2_ channel. This configuration enables a smoother and more uniform electron injection pathway. As a result, carrier injection is significantly improved, contributing to enhanced device performance. Figure 4c shows representative transfer characteristics of both device types. Gr–MoS_2_ FETs (red) exhibit significantly enhanced on-state current and improved subthreshold swing (SS) compared to conventional MoS_2_ FETs (black), reflecting more efficient carrier injection and better gate control. In addition, both device types maintain high on/off current ratios exceeding 10^6^, indicating that the introduction of graphene contacts does not degrade the switching characteristics. To quantitatively compare carrier transport, the field-effect mobility values for both FET types were extracted using maximal *g*_m_ and statistically analyzed. As shown in Figure 4d, Gr–MoS_2_ FETs exhibit a significantly higher average field-effect mobility of 3.9 cm^2^ V^−1^s^−1^, compared to 1.1 cm^2^ V^−1^s^−1^ for conventional MoS_2_ FETs. The performance enhancement is primarily attributed to the use of graphene as the source and drain, which effectively replaces the conventional metal–MoS_2_ contacts and leads to a significant reduction in contact resistance, thereby enabling more efficient charge injection into the MoS_2_ channel. Figure 4e presents a comparative analysis of the resistance components for both devices. All values were extracted using the Y-function method [43], which relates the drain current ($I_{DS}$) and transconductance ($g_{m}=\frac{\partial I_{DS}}{\partial V_{GS}}$) as follows:

$$Y=\frac{I_{DS}}{\sqrt{g_{m}}}=\sqrt{{µ}_{0}C_{ox}\frac{W}{L}V_{DS}}\cdot (V_{GS}-V_{TH}),$$

where ${µ}_{0}$ is the intrinsic mobility excluding contact resistance, and $C_{ox}$ is the gate dielectric (SiO_2_) capacitance per unit area. In addition, W and L denote the channel width and length, respectively, while $V_{DS}$, $V_{GS}$, and $V_{TH}$ correspond to the drain-to-source voltage, gate-to-source voltage, and the threshold voltage. The extracted resistance components at V_G_ = 40 V clearly show that the contact resistance ($R_{c}$) in Gr–MoS_2_ FETs is significantly lower than that in conventional MoS_2_ FETs. Specifically, the average $R_{c}$ value for Gr–MoS_2_ FETs is 20.5 kΩ, compared to 85.8 kΩ for MoS_2_ FETs with metal contacts. In contrast, the channel resistance ($R_{ch}$) remains relatively comparable between the two device types. This pronounced reduction in contact resistance for the Gr-MoS_2_ FETs confirms that the enhancement in mobility and overall device performance is primarily attributed to the improved contact properties enabled by the use of graphene as source and drain. This comparative analysis underscores the critical role of contact engineering in 2D material-based devices and demonstrates that replacing conventional metal contacts with graphene is an effective strategy for enhancing transport performance.

## 4. Conclusions

In this work, we successfully fabricated and characterized Gr–MoS_2_ lateral heterostructure FETs, in which monolayer graphene functions directly as the source and drain. The heterostructure was synthesized via an edge-selective CVD process that enables lateral stitching of bilayer MoS_2_ onto patterned graphene, forming a chemically bonded and well-defined interface. Electrical measurements revealed that the Gr–MoS_2_ FETs exhibit superior performance compared to conventional MoS_2_ FETs. The average field-effect mobility increased from 1.1 to 3.9 cm^2^ V^−1^s^−1^, while the contact resistance decreased markedly from 85.8 kΩ to 20.5 kΩ at V_G_ = 40 V. This study highlights the effectiveness of graphene as a contact material for 2D semiconductors and demonstrates that lateral heterostructure engineering offers a scalable and CMOS-compatible route to reduce contact resistance and enhance carrier injection. The Gr–MoS_2_ platform presented here holds strong potential for future high-performance, large-area 2D electronic applications.

## Data Availability

The original contributions presented in this study are included in the article. Further inquiries can be directed to the corresponding author.
